# Bare and boron-doped cubic silicon carbide nanowires for electrochemical detection of nitrite sensitively

**DOI:** 10.1038/srep24872

**Published:** 2016-04-25

**Authors:** Tao Yang, Liqin Zhang, Xinmei Hou, Junhong Chen, Kuo-Chih Chou

**Affiliations:** 1 State Key Laboratory of Advanced Metallurgy, University of Science and Technology Beijing, Beijing 100083, China; 2 School of Material Science and Engineering, University of Science and Technology Beijing, Beijing 100083, China

## Abstract

Fabrication of eletrochemical sensors based on wide bandgap compound semiconductors has attracted increasing interest in recent years. Here we report for the first time electrochemical nitrite sensors based on cubic silicon carbide (SiC) nanowires (NWs) with smooth surface and boron-doped cubic SiC NWs with fin-like structure. Multiple techniques including scanning electron microscopy (SEM), transmission electron microscopy (TEM), X-ray diffraction (XRD), X-ray photoelectron spectroscopy (XPS) and electron energy loss spectroscopy (EELS) were used to characterize SiC and boron-doped SiC NWs. As for the electrochemical behavior of both SiC NWs electrode, the cyclic voltammetric results show that both SiC electrodes exhibit wide potential window and excellent electrocatalytic activity toward nitrite oxidation. Differential pulse voltammetry (DPV) determination reveals that there exists a good linear relationship between the oxidation peak current and the concentration in the range of 50–15000 μmoL L^−1^ (cubic SiC NWs) and 5–8000 μmoL L^−1^ (B-doped cubic SiC NWs) with the detection limitation of 5 and 0.5 μmoL L^−1^ respectively. Compared with previously reported results, both as-prepared nitrite sensors exhibit wider linear response range with comparable high sensitivity, high stability and reproducibility.

Functional application of wide bandgap compound semiconductors has shown an increased interest in the recent years because of their unique electrical and thermal properties. Silicon carbide (SiC) belongs to the class of wide band gap semiconductors with band gap energy varying from 2.4 to 3.2 eV depending on the polytype^1^,^2^. It possesses high thermal conductivity, on par with copper at room temperature^3^,^4^. Its Young’s modulus is higher than that of Si and its high breakdown field about 2 MV cm^−1^ is double than that of Si^5^. In view of the conductivity of SiC, the intrinsic carrier concentration of SiC is ~10^16^–10^18 ^cm^−3^, while for Si is ~10^10 ^cm^−3^. It is more than 6 orders of magnitude higher than that of Si^6^. Compared with other semiconductors, SiC’s wide band-gap increases its sensing capabilities of electrochemistry. Since SiC nanowires possess special micro morphology and excellent electrical properties, they have been applied in many fields, such as field-effect transistors^7^,^8^,^9^, microwave absorption^10^, photocatalysts^11^,^12^ and piezoresistance^13^,^14^. Some recent research work indicates that SiC electrode behaves similarly to an oxidation-reduction indicator material such as gold and platinum and shows a wide potential window, i.e. +1.4 V to −1.2 V vs Ag/AgCl electrode^15^,^16^. In addition, SiC electrodes could show excellent selective electrocatalytic behavior with high sensitivity, excellent catalytic activity, short response time and long term stability^17^,^18^,^19^,^20^. These findings have led to the construction of different SiC electrodes mainly for the biosensor application^15^,^16^,^17^,^18^,^19^,^20^,^21^,^22^,^23^. Therefore, Oliveros *et al*.^24^ pointed out that SiC could be a versatile material for functional applications.

Environmentally important compounds such as nitrites have attracted increasing attention of analytical chemists and electrochemist in recent years due to their potential toxicity^25^. The presence of nitrite in groundwater and atmosphere is an essential precursor in the formation of nitrosamines, many of which have been proven to be powerful carcinogens^26^,^27^. Therefore, the World Health Organization (WHO) has fixed the maximum limit of 3 mg L^−1^ (65.22 μmoL L^−1^) for nitrite in drinking water^28^. Among the various determination methods, electrochemical methods have often been employed for detection of nitrite owing to the rapid response, cheaper, safer and simpler use^29^,^30^. The electrochemical determination of nitrite at traditional electrodes, such as platinum electrode and glassy carbon electrode (GCE)^31^,^32^ has been developed. However, the direct electroreduction/oxidation of nitrite ions requires high overpotential (0.8 V) at bare electrode surfaces^32^. In addition, the determination of nitrite at bare electrode always suffers from the interference from other compounds^33^. Therefore, various modification electrodes such as metallophthalocyanines and metalloporphyrins, metal nanoparticles, series of inorganic materials and enzyme modified electrodes have been proposed for nitrite sensing^34^,^35^. Two-dimensional (2D) nanostructures such as nanowires also favor the charge transfer, which in turn enhance the electrochemical activity to some degree. Therefore WO_3_ nanowires^36^, uniform β-MnO_2_ nanorods^37^, and Pt–Ru nanowire^38^ etc. have been adopted as nitrite sensors.

Considering electrochemical determination of nitrite is an electrocatalytic process, SiC NWs could be applied as a nitrite sensor based on their special micro morphology and excellent electrical properties. While the research on it is less reported except that Salimi *et al*.^21^ fabricated the SiC nanoparticles/amine terminated ionic liquid modified glassy carbon electrode to determine nitrite. Herein the fabrication of nitrite sensor based on SiC NWs is reported for the first time. In addition, the method of doped SiC for instance B-doping is employed to improve the electrochemical sensitivity^39^ Compared with the nitrite sensor reported in the literature, the advantages of the SiC-based nitrite sensor are as following: (1) the electrode fabrication process is a relatively simple one without using any specific electron transfer mediator; (2) Both modified cubic SiC NWs nitrite sensors exhibit larger linear response range while with comparable higher sensitivity than the reported results, such as metal modified, organics modified, oxide modified, non-oxide modified and SiC nanoparticles modified GC nitrite sensors. They also show high stability and satisfactory reproducibility.

## Results

### Phase and Morphology Characterization

XRD patterns of the as-prepared samples are shown in Fig. S1a. Three strong diffraction peaks at 2*θ* = 35.8°, 60° and 72°appeared in SiC NWs sample, which correspond to the (111), (220) and (311) facets of cubic SiC (JCPDS card no. 73-1665). As for B-doped SiC NWs, B doping does not change the phase structure of cubic SiC as shown in Fig. S1a. However, the shift in 2*θ* values (the inset of Fig. S1a) indicates B has partly substituted Si sites in SiC crystal because the radius of boron atom (0.095 nm) is smaller than silicon atom (0.134 nm)^40^. To confirm the existence of B element in B-doped cubic SiC NWs, EELS was carried out. The peak centered at 188 eV corresponds to B element and the peak at 284 eV corresponds to C element (Fig. S1b). This indicates that B has doped into cubic SiC in the experiment. The B/Si molar ratio, as determined by XPS (Fig. S1c) is 0.066, which is close to the theoretical content of Si_15_BC_16_^11^. It should be pointed out that the stacking faults (SF)^41^ in both SiC NWs and B-doped SiC NWs are relatively less. This is beneficial to electron transfer.

**Figure 1:**
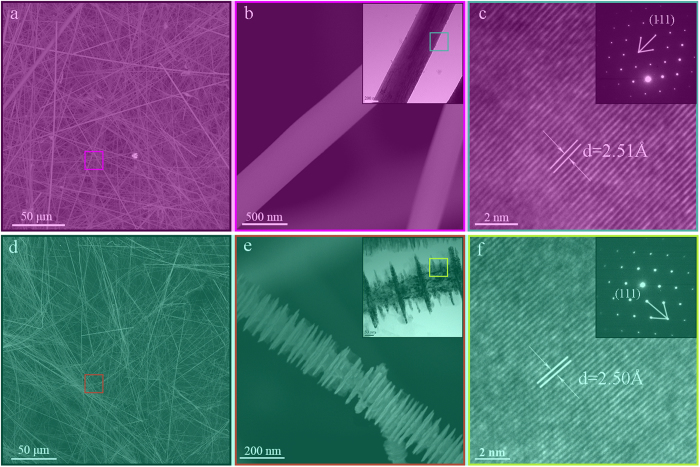
SEM and TEM images of the as-prepared cubic SiC (**a**–**c**) and B-doped cubic SiC (**d**–**f**).

The morphology and microstructure of the as-prepared samples are revealed by SEM and TEM techniques (Fig. 1). The SEM images (Fig. 1a,b) show that the typical cubic SiC NWs possess a smooth surface and the average diameter is about 80 nm. From the HRTEM image and the SAED pattern in Fig. 1c, the NWs possess a homogeneous crystalline structure with a fringe spacing of 2.51 Å, which is characteristic of cubic SiC. Compared with the typical morphology of SiC NWs (Fig. 1a–c), B-doped SiC NWs (Fig. 1d–f) possess fin-like microstructure containing inner core stems and outer fins. TEM images (the inset of Fig. 1e) indicate that the diameter of the outer fins is about 100–200 nm and the inner core stem is about 80 nm. The thickness of the fins is 10–20 nm. The HRTEM image (Fig. 1f) reveals that the lattice spacing is 2.50 Å, which is smaller than the standard value of SiC (Fig. 1c). The main reason is that the substitution of smaller B (0.095 nm) at Si (0.134 nm) and leads to the distortion of lattice. The corresponding SAED pattern inset Fig. 1f shows the nature of the nanowire is single crystal.

Considering the electrochemistry of conductive nanowires is dependent on the morphology, such as size, density, spacing in between wires, etc. From SEM and TEM images, the typical cubic SiC NWs possess a smooth surface and the average diameter is about 80 nm and B-doped cubic SiC NWs is fin-like nanowires composed of inner core stems (the diameter of 80 nm) and outer fins (the diameter of 100–200 nm). All of these SiC NWs are single crystals, which is beneficial to electron transfer. In addition, the SF in both SiC NWs and B-doped cubic SiC NWs are relatively less (Fig. S1), which is also beneficial to electron transfer.

### Electrochemical characterization

**Figure 2:**
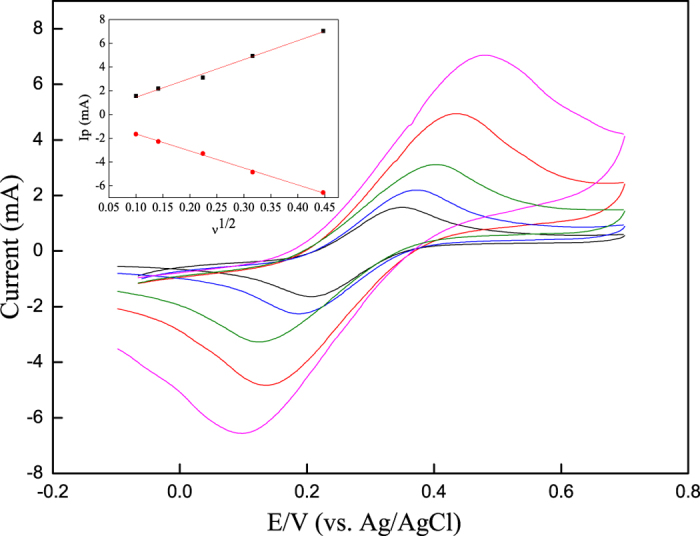
CV curves at cubic SiC NWs electrode in 0.5 moL·L^−1^ KCl containing 0.01 moL·L^−1^ [Fe(CN)_6_]^3−^^/4−^ at the scan rates of 10, 20, 50, 100, 200 mV·s^−1^ (from inner to outer). The inset is the plot of anodic/cathodic peak currents versus the square root of scan rate.

By using a 0.01 moL L^−1^ ferrocyanide and ferricyanide couple (1:1) as the redox probe and employing cubic SiC NWs or B-doped SiC NWs as the working electrode, the charge-transfer rates at the solution/electrode interface in a 0.5 moL L^−1^ KCl solution were measured at the scan rate of 0.01–0.2 V s^−1^. As for SiC NWs electrode (Fig. 2), the anodic and cathodic peaks associating with the oxidation and reduction of the ferricyanide-ferrocyanide couple obviously appear at the SiC-solution interface, respectively. Similar phenomena also appear at B-doped cubic SiC-solution interface as shown in Fig. S2. The anodic and cathodic peak current both increase linearly with the square root of scan rates as shown in the inset of Fig. 2 and Fig. S2, exhibiting that the electrode reactions are diffusion controlled. According to Laviron^42^, the electron transfer rate constant is calculated to be 2.53 × 10^−2 ^cm s^−1^ for cubic SiC NWs electrode and 3.61 × 10^−2 ^cm·s^−1^ for B-doped cubic SiC NWs electrode. Accordingly, it can be seen that the ratio of the anodic/cathodic peak current values is close to unit, indicating a quasi-reversible process.

To better understand the electrochemical sensing mechanism, EIS analysis was used to measure the charger transfer resistance at the electrode | electrolyte interface^43^. EIS was performed in 0.5 moL L^−1 ^KCl containing 0.01 moL L^−1 ^Fe(CN)_6_^3−/4−^ as the supporting electrolyte. Fig. S3 represents the Nyquist plots of both cubic SiC NWs and B-doped cubic SiC NWs electrodes. It is known that the diameter of the semicircle is a direct representation of the charge transfer resistance (Rct). By comparison, the value of Rct for B-doped cubic SiC NWs, i.e. 5.33 Ω, is much lower than that of cubic SiC NWs electrode, 26.83 Ω, demonstrating that B-doped cubic SiC NWs electrode has a smaller charge transfer resistance and thus provides facile electron pathways between the electrode and electrolyte.

### Electrochemical determination of nitrite

**Figure 3:**
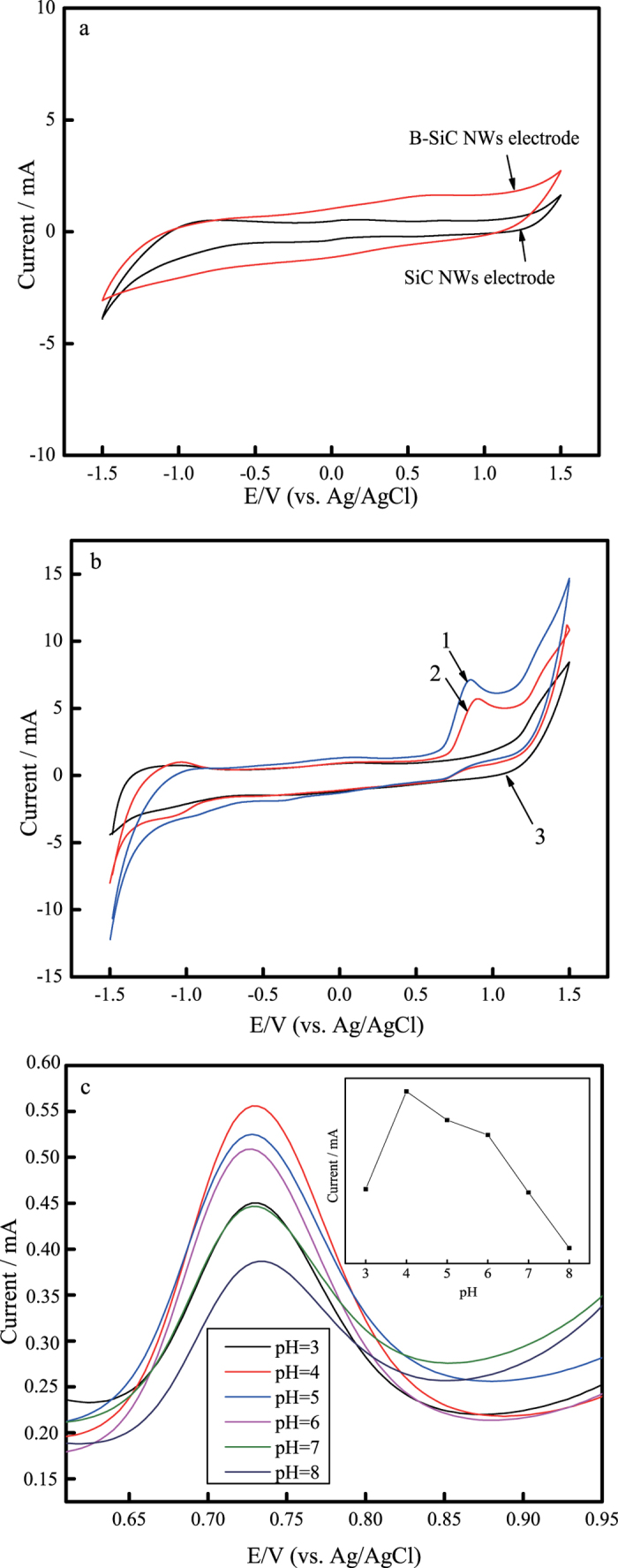
(**a**) CV curves at cubic SiC NWs electrode (black) and B-doped cubic SiC NWs electrode (red) in 0.1 moL·L^−1^ PBS (pH 4.0) with *v* = 0.05 V·s^−1^; (**b**) CV curves at B-doped cubic SiC NWs electrode (1), cubic SiC NWS electrode (2) and the bare Ti electrode (3) in 0.1 moL·L^−1^ PBS (pH 4.0) containing 0.01 mmoL·L^−1^ nitrite; (**c**) DPV curves at B-doped cubic SiC NWs electrode in 0.1 moL·L^−1^ PBS containing 0.01 moL·L^−1^ nitrite with different pH.

Considering SiC as electrode should exhibit a wide potential window due to its special electric property and excellent chemical and thermal stability^44^, CVs of cubic SiC NWs electrode and B-doped cubic SiC NWs electrode were carried out in 0.1 moL L^−1^ phosphate buffer solutions (PBS, pH 4.0) as shown in Fig. 3a. Since the surface of SiC tends to absorb oxygen^45^, the solution was degassed using nitrogen for at least 15 min prior to data acquisition. The redox response corresponds to the cathodic and anodic peaks of water at SiC NWs electrode. It can be seen that both the electrodes have a wide potential window from −1.5 to 1.5 V. It is well known that the direct electroreduction/oxidation of nitrite ions requires high overpotential, i.e. 0.8 V at bare electrode surfaces^21^,^46^. Therefore both cubic SiC NWs electrode and B-doped cubic SiC NWs electrode ensure the electrochemical detection of nitrite in this work.

The oxidation of nitrite at the different working electrodes, i.e. B-doped cubic SiC NWs electrode (1), cubic SiC NWS electrode (2) and the bare Ti electrode (3) in 0.1 moL·L^−1^ PBS (pH 4.0) containing 0.01 mmoL·L^−1^ nitrite were carried out using CV methods. The scan rate was 50 mV·s^−1^. As shown in Fig. 3b, both the electrodes, i.e. modified with B doped cubic SiC NWs (1) and cubic SiC NWs electrode (2) exhibit an obvious oxidation peak at 0.8 V, indicating that the SiC NWs electrode is able to catalyze the electrochemical oxidation reaction of nitrite. By comparison, a remarkably larger peak current was obtained at B-doped cubic SiC NWs electrode (7.22 mA), indicating better catalytic ability. As for the bare Ti electrode (3), there is no obvious oxidation peaks, indicating that the bare Ti electrode is unable to catalyze the electrochemical oxidation of nitrite.

The influence of solution pH on the electrochemical response of nitrite (1 mmoL·L^−1^) at B-doped cubic SiC NWs electrode was examined by recording DPVs in PBS (pH 3.0–8.0) at a scan rate of 50 mV·s^−1^. As shown in Fig. 3c, the oxidation peak current of nitrite is greatly influenced by solution pH and the peak currents gradually decreased with pH in the range of 4.0–8.0. The maximum peak currents were obtained at the pH of 4.0. Since the p*Ka* of HNO_2_ is 3.3, most nitrite ions are protonated in the acidic solutions. It is possible that protonation is involved in the catalytic reaction, so the catalytic peak current increases with the decrease of solution pH^47^. The influence of solution pH on the electrochemical response of nitrite (1 mmoL·L^−1^) at cubic SiC NWs electrode was also examined and the result show that the maximum peak currents were obtained at the pH of 4.0. In addition, the results show that the peak potential for nitrite oxidation is not affected by pH value of the solution. The possible reason is attributed to a kinetically controlled oxidation process, i.e. a proton independent catalytic step^48^.

The influence of different scan rates on the oxidation behavior of nitrite at cubic SiC NWs electrode and B-doped cubic SiC NWs electrode respectively was investigated by LSV. Fig. S4a and b show the LSV curves for the anodic oxidation of nitrite at cubic SiC NWs electrode and B-doped cubic SiC NWs electrode at various scan rates in the presence of 1 mmoL·L^−1^ nitrite in PBS (pH = 4.0). It is clear that the oxidation peak current both increases continuously with the increase of the scan rate, while the peaks potential shift positively. Moreover, there is a linear relationship between the peak current (*Ip*) and the square root of scan rate (ν^1/2^) as showed in Fig. S4c,d. For cubic SiC NWs electrode, the fitted regression equation can be expressed as follows:


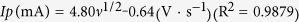


For B-doped cubic SiC NWs electrode, the fitted regression equation can be expressed as following:


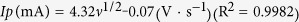


The above both indicate the kinetics of the overall process is controlled by diffusion. In addition, the oxidation peaks potential (*Ep*) all shift to more positive potentials with the increase of the scan rate. This leads to a linear relationship between *Ep* and log*ν* as shown in Fig. S4e,f. This confirms the irreversibility of the electrocatalytic oxidation process of nitrite. The linear relationship can be expressed respectively as following:


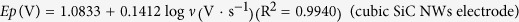



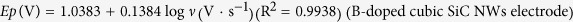


From this straight line, the slope and the intercept can be given as 0.1412 and 1.0833 for cubic SiC NWs electrode and 0.1384 and 1.0383 for B-doped cubic SiC NWs electrode. For an irreversible process, the peak potential (*Ep*) could be present by the equation^49^ as following:


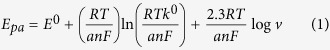


where *α* is the electron transfer coefficient, *n* is the number of transfer electron, *R, T* and *F* have their usual meanings. Thus, *αn* was calculated to be 0.4183 (cubic SiC NWs electrode) and 0.4267 (B-doped cubic SiC NWs electrode). Generally, *α* is assumed to be 0.5 in a totally irreversible electrode process. Therefore, the number of transfer electron (*n*) in oxidation of nitrite was about 1, which is in agreement with previous literature reports^34^.

From above experimental results, it can be seen that the overall reaction occurred at SiC NWs based electrodes toward nitrite determination can be expressed as follows:


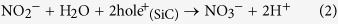


The oxidation reaction process can be derived through an electrocatalytic mechanism into the following two steps. In the first step, NO_2_^−^ is oxidized to NO_2_ (Eq. (3)) and in the second step NO_2_ oxidized to NO_3_^−^ (Eq. (4))


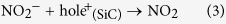



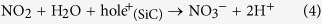


**Figure 4:**
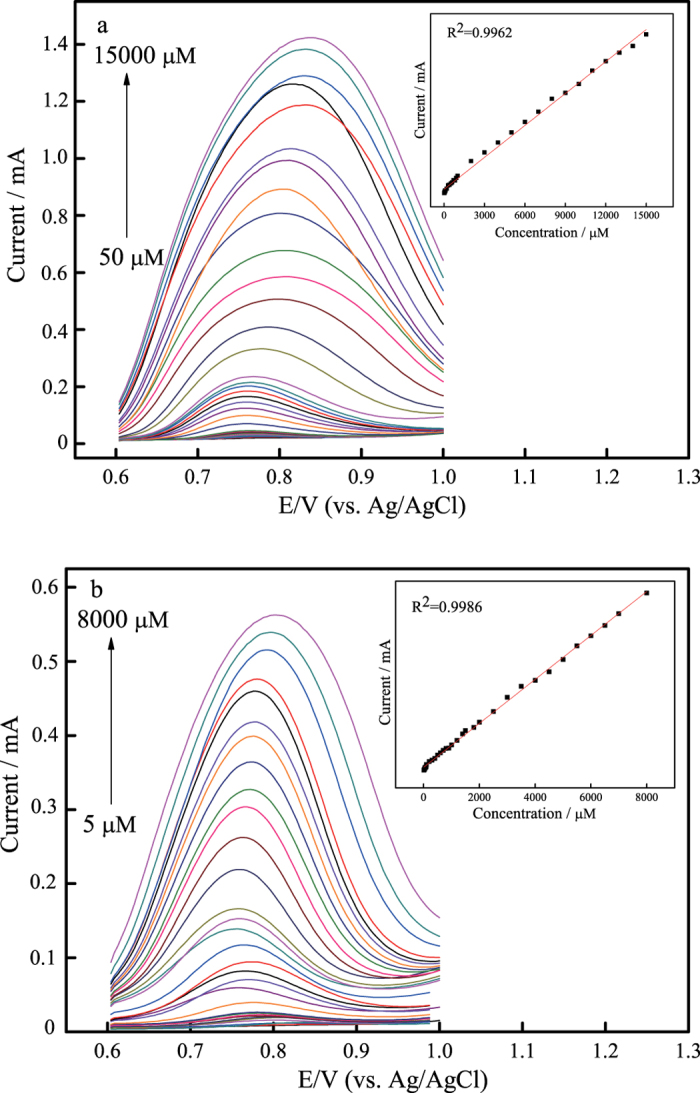
DPV recordings of nitrite at (**a**) cubic SiC NWs electrode and (**b**) B-doped cubic SiC NWs electrode in PBS (0.1 moL·L^−1^) with different nitrite concentrations, *v* = 0.01 V·s^−1^.

DPV is a pulse technique which allows much higher sensitivity than conventional sweep techniques when detecting very low concentrations of an analyte. In this work, DPV was performed to examine the sensitivity of cubic SiC NWs electrode and B-doped cubic SiC NWs electrode toward nitrite in static solutions. The DPVs were recorded by sweeping the potential between 0.6 and 1.0 V at amplitude of 0.025 V, a step potential of 0.05 V and a scan rate of 10 mV·s^−1^. Fig. 4a,b depict the DPV curves of cubic SiC NWs electrode and B-doped cubic SiC NWs electrode in PBS solution with different nitrite concentrations. In the inset of Fig. 4a,b both DPV curves show the well-defined and stable anodic oxidation peak current curves for nitrite, demonstrating that both electrodes might provide a good electrocatalytic activity toward nitrite. As for cubic SiC NWs electrode, the DPV current response increase linearly with nitrite concentration increasing in the range of 50–15000 μmoL·L^−1^ as shown in the inset to Fig. 4a. The fitted regression equation can be expressed as:


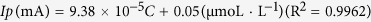


Similarly, the linear relationship between DPV current response and nitrite concentration for B-doped cubic SiC NWs electrode in the range of 5–8000 μmoL·L^−1^ can be expressed as follows:


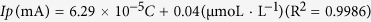


The detection limitation for nitrite is 5 μmoL·L^−1^ with the sensitivity of 0.0938 μA·μmoL^−1^ for cubic SiC NWs electrode. As for B-doped cubic SiC NWs electrode, the detection limitation for nitrite is 0.5 μmoL·L^−1^ with the sensitivity of 0.0629 μA·μmoL^−1^.

As for the electrochemical performance of SiC NWs based nitrite sensor, the comparison with the reported result in the literature, such as metal, organics, oxide, carbon modified composites and SiC modified GC, is listed in Table 1. It can be seen that SiC NWs based electrode exhibits larger linear response range while with comparable higher sensitivity^21^,^34^,^36^,^37^,^50^,^51^,^52^,^53^,^54^,^55^,^56^,^57^, demonstrating the promising application of modified SiC electrode for nitrite determination. At the same time, it should be pointed out that the sensor preparation in this work is also relatively simpler than that reported in the literature.

**Figure 5:**
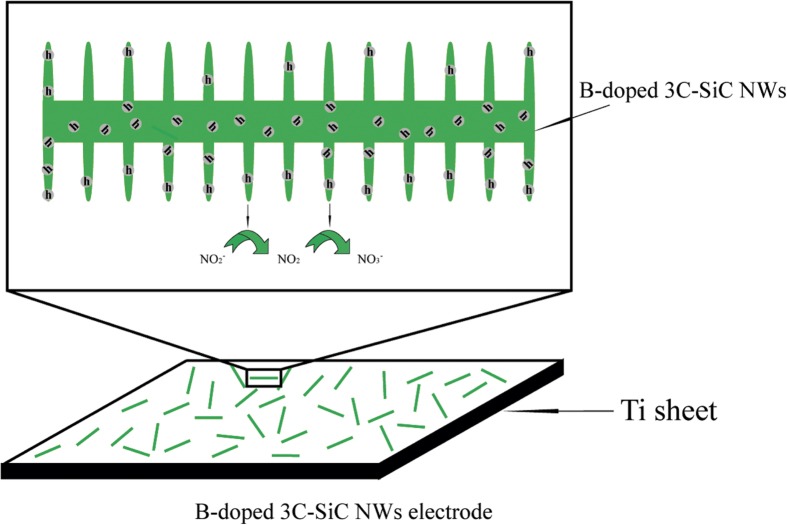
The schematic illustration of electrochemical detection of nitrite based on B-doped cubic SiC NWs electrode.

In view of cubic SiC NWs electrode, the good performance toward nitrite determination is mainly attributed to its unique electronic properties such as good conductivity, facilitating electron transfer. As for the outstanding electrochemical performance of B-doped cubic SiC NWs, the possible mechanism is shown in Fig. 5 and three factors are attributed. First, more positive holes, i.e. hole^+^_(SiC)_ caused by the transformation from intrinsic semiconductor to p-type semiconductor after B doping are produced. The positive holes prone to oxidize nitrite. Second, the conductive ability of SiC is enhanced after B doping^9^,^58^ and thus electron can be transferred efficiently by NWs^11^. Third, the special fin-like microstructure increases the aqueous suspension contact area. This can be efficiently transduced of the surface adsorption of ions into a change of electronic conductivity.

### Amperometric detection of nitrite

**Figure 6:**
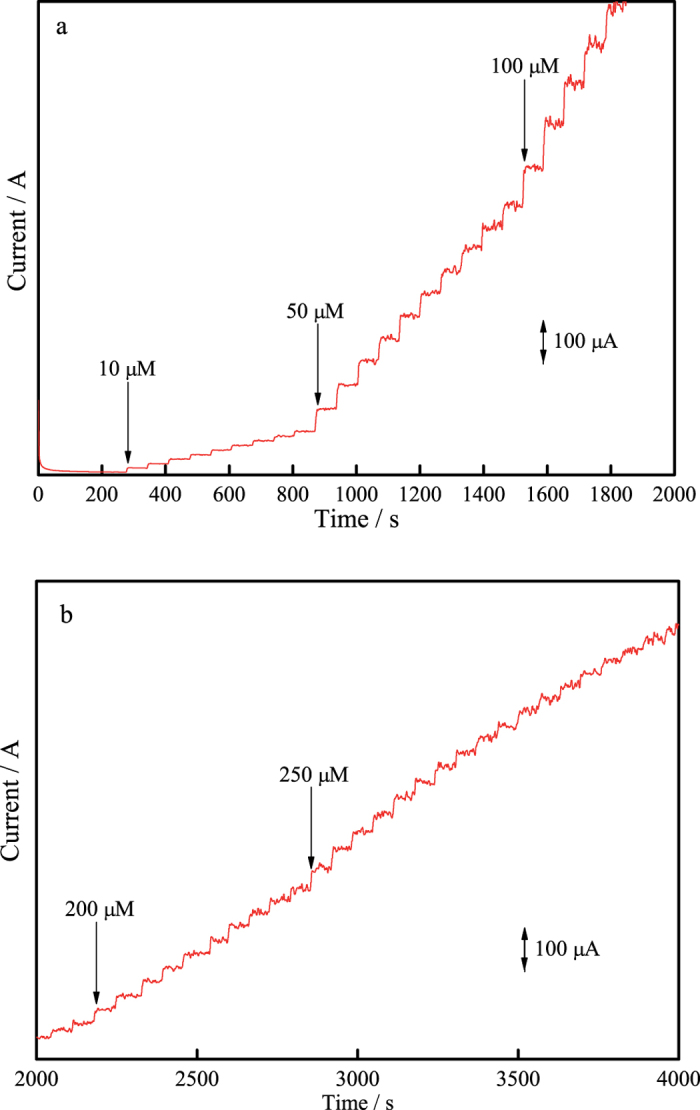
The current response to low concentrations of nitrite on cubic SiC NWs electrode in PBS (0.1 moL·L^−1^, pH = 4.0).

A typical current-time response curve for the successive addition of nitrite to stirred 0.1 moL·L^−1^ PBS (pH = 4.0) at the applied potential 0.8 V is shown in Fig. 6. The results clearly indicate that both cubic SiC NWs electrode (Fig. 6a,b) and B-doped cubic SiC NWs electrode (Fig. S5a,b) respond to the increasing nitrite concentration quickly and sensitively. The current response time of both electrodes is less than 2 s after the addition of nitrite.

### Reproducibility and stability

In order to investigate the stability of SiC based electrode, electrochemical experiments were repeatedly performed 20 times on cubic SiC NWs electrode and B-doped cubic SiC NWs electrode respectively in the PBS containing 1 mmoL·L^−1^ nitrite. The relative standard deviation (% R.S.D.) is 4.0% (cubic SiC NWs electrode) and 3.5% (B-doped cubic SiC NWs electrode). When stored in PBS (pH = 4.0) at 4 °C, the current response almost stabilized for at least two weeks and no obvious fading in response to nitrite is observed after 30 days. The preparation of five electrodes with the same method shows an acceptable reproducibility with a RSD of 5.6% (cubic SiC NWs electrode) and 5% (B-doped cubic SiC NWs electrode) for the current response at 0.1 PBS containing 1 mmoL·L^−1^ nitrite.

### Anti-interference performance

**Figure 7:**
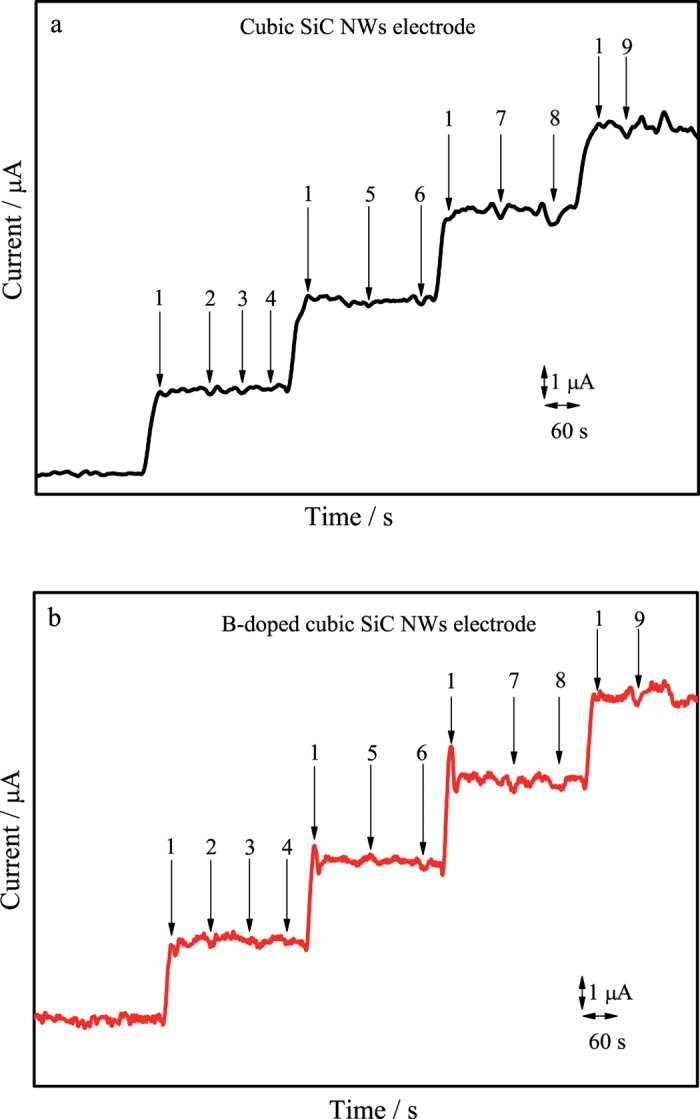
The amperometric response at the cubic SiC NWs electrode (**a**) and B-doped cubic SiC NWs electrode (**b**) for nitrite (1) in the presence of 100-fold: NaNO_3_ (2), CuSO4 (3), NaCl (4), K_2_SO_3_ (5), FeCl_2_ (6) NaOH (7), urea (8), glucose (9).

The possible interference for the nitrite detection was investigated by adding some inorganic ions and organic compounds, which may coexist with nitrite in real samples. As shown in Fig. 7, both electrodes exhibit well defined amperometric response towards each addition of nitrite (1). However, there was no significant response observed for each 100-fold excessive addition of ions or agents including both non-reducing agents and reducing ions. For instance NaNO_3_ (2), CuSO_4_ (3), NaCl (4), K_2_SO_3_ (5), FeCl_2_ (6), NaOH (7), urea (8) and glucose (9). The results clearly demonstrate that nitrite can be selectively detected by SiC NWs based electrode.

### Application in practical analysis

To illustrate the application of the electrodes in practical analysis, both electrodes are employed for nitrite assay by the standard addition method in tap water sample. The tap water sample was prepared according to the reported work by Qin *et al*.^59^ and the results are summarized in Table S1. It can be clearly observed that a good recovery is obtained, suggesting this proposed method can be successfully applied for the detection of nitrite in real sample.

## Conclusions

The electrochemical nitrite sensors based on cubic SiC NWs with smooth surface and fin-like B doped SiC NWs are reported in this work for the first time. Compared with the reported result in the literature, such as metal, organics, oxide, carbon modified composites and SiC modified GC nitrite sensors, the electrochemical performance of SiC NWs based electrode exhibits larger linear response range while with comparable higher sensitivity. The good performance toward nitrite determination is mainly attributed to the special microstructure and electronic properties of SiC. In addition, both SiC-based electrodes show high stability, good repeatability and anti-interface ability. The good recovery obtained in the real water sample studies reveals the promising practical utility of the proposed sensor based on wide bandgap semiconductor.

## Methods

### Materials

Gangue (SiO_2_ > 99%), boric oxide (≥98%), carbon black, hydrofluoric acid (HF), polyvinylidene fluoride (PVDF), N-methyl-2-pyrrolidone (NMP) and sodium nitrite were supplied by Sinopharm Chemical Reagent Beijing Co., Ltd (SCRB). Ferrocyanide and ferricyanide were supplied by Aladdin. The PBS with different pH values were prepared using Na_2_HPO_4_ and NaH_2_PO_4_. Argon gas and nitrogen gas was supplied by Haipu Gas Co., Ltd. Deionized water was used in all experiments.

### Preparation of SiC based NWs. Materials

Cubic SiC NWs in large scale were fabricated by one-step thermal reduction method using gangue and carbon black as raw material^60^. Gangue and carbon black with a mole ratio of 1:3 were ball mixed. The resulting mixture were put in ceramic boat and placed in the hot zone of a furnace equipped with carbon as an inner lining. Before heating, the furnace was evacuated and then high purity argon was introduced at a constant gas flow. The pressure was maintained at 1 atm throughout the whole experiment. The reaction was took place at 1500 °C for 2 h in flowing argon. Finally the furnace was cooled naturally to room temperature. The obtained whiskers were washed with 10% hydrofluoric acid (HF) for 1 h to remove the residual silica.

B-doped cubic SiC NWs were prepared in a similar way as cubic SiC NWs except the raw materials used^11^. Gangue, boric oxide and carbon black with the mole ratio of 1:32:96 were used as raw materials. The same experimental steps were adopted to prepare B-doped cubic SiC NWs.

### Fabrication of the SiC based electrodes

Cubic SiC NWs electrode was prepared by mixing cubic SiC NWs and PVDF with the mass ratio of 8:2 in NMP and the stable slurry was coated on a Ti sheet with the coating mass of ~0.1 mg (1 × 1 cm). B-doped cubic SiC NWs electrode was prepared from the same method. The above electrodes were heated at 90 °C for 3 h to evaporate the solvent for electrochemical detection. All of SiC NWs were firmly stick on the titanium plate, in which electrical charges can directly transfer to the titanium plate. So spacing in between wires should not affect the electrical conductivity.

### Instrumentation

The obtained products were characterized using X-ray diffraction (XRD) with Cu Kα radiation (λ = 1.54178 Å) (XRD, TTRIII, Rigaku). The accelerating voltage and the applied current were 40 kV and 40 mA, respectively. The chemical states of B-doped SiC nanowires samples were determined by X-ray photoelectron spectroscopy (XPS) in a VG Multilab 2009 system (UK) with a monochromatic Al Kα source and a charge neutralizer. The surface morphologies of the products were examined by a field emission scanning electron microscope (FEI-SIRION, operated at 5 kV). Transmission electron microscopy (TEM) and high-resolution transmission electron microscopy (HRTEM) images were collected by a JEOL model JEM 2010 EX microscope, using an accelerating voltage of 200 kV. EELS experiments were performed using a JEOL 3000F microscope equipped with a Gatan GIF2000 1 K Phosphor spectrometer system operating at 300 kV.

### Electrochemical measurement

The electrochemical measurements were carried out on a CHI660E electrochemistry working station. A conventional three-electrode cell was used with a silver chloride electrode as the reference, a platinum wire as the counter and cubic SiC NWs electrode or B-doped cubic SiC NWs electrode as the working electrode. Electrochemical measurements include cyclic voltammogram (CV), electrochemical impedance spectroscopy (EIS), linear sweep voltammetry (LSV), DPV, current-time plot and amperometric response were carried out. The electrochemical property of working electrodes were performed in solution of KCl containing ferrocyanide and ferricyanide with the scan rate range from 10 to 200 mV·s^−1^. CV, LSV, DPV and I-t of the working electrodes were performed in the solution of phosphate buffer solution (PBS) containing nitrite. The scan rate of CVs is 0.05 V·s^−1^ and 10–200 mV·s^−1^ for LSV. DPV experiments were performed with amplitude of 50 mV, pulse width of 0.2 s and pulse period 0.5 s. All experiments were carried out at about 25 ± 1 °C. All solutions were prepared using reagent grade chemicals in deionized water. The solution pH was kept at 4.0.

## Additional Information

**How to cite this article**: Yang, T. *et al*. Bare and boron-doped cubic silicon carbide nanowires for electrochemical detection of nitrite sensitively. *Sci. Rep.* **6**, 24872; doi: 10.1038/srep24872 (2016).

## Supplementary Material

Supplementary Information

## Figures and Tables

**Table 1:** Comparison of electrochemical performance for the determination of nitrite using different electrodes.

Electrode material	Linear range (μmoL·L^−1^)	Detection limit (μmoL·L^−1^)	Ref.
NH2-IL/SiCnp/GC	0.05–0.35	0.02	21
Nano-Au/P3MT/GCE	10–1000	2.3	34
WO_3_ nanowires	1–4200	0.28	36
β-MnO_2_ nanorods	0.29–26.09	0.29	37
Au–G–PANI/GCE	0.1–205.8	0.01	50
Fe-HNPs	9.0–3000	2.6	51
Cu_nano_/CNTs/CS	0.1–2500	0.024	52
PDDA/P_2_W_17_V-CNTs	0.05–2130	0.0367	53
G_4_-NH_4_/MWCNT	5–50	2	54
Fe_2_O_3_/rGO	0.05–780	0.015	55
Fe_3_O_4_/rGO	1–92	0.3	56
SiO_2_/C/MnPc	0.79–15.74	0.02	57
Cubic SiC NWs	50–15000	5	This work
B-doped cubic SiC NWs	5–8000	0.5	This work
