# Supplementary material for: Bare and boron-doped cubic silicon carbide nanowires for electrochemical detection of nitrite sensitively
Source: Sci Rep. 2016 Apr 25;6:24872. doi: 10.1038/srep24872 (PMC4843007; doi:10.1038/srep24872)
Supplement: Supplementary Information [file srep24872-s1.doc]

Supplementary Information

**Bare and boron-doped cubic silicon carbide nanowires for electrochemical detection of nitrite sensitively**

Tao Yang^1^, Liqin Zhang^1^, Xinmei Hou^2^*, Junhong Chen^2^, Kuo-Chih Chou^1^

^1^ State Key Laboratory of Advanced Metallurgy, University of Science and Technology Beijing, Beijing 100083, China

^2^ School of Material Science and Engineering, University of Science and Technology Beijing, Beijing 100083, China

*Fax: +86 10 6233 2570; Tel: +86 10 6233 2570; Email:* [*houxinmei@ustb.edu.cn*](mailto:houxinmei@ustb.edu.cn)

**Figure [S1]** (a) XRD pattern of cubic SiC and B-doping cubic SiC. (b) EELS of B-doped cubic SiC. (c) XPS spectra of B-doping cubic SiC.

**Figure [S2]** CV curves at B-doped cubic SiC NWs electrode in 0.5 moL·L^-1^ KCl with 0.01 moL·L^-1^ [Fe(CN)_6_]^3−/4−^ at the scan rates of 10, 20, 50, 100, 200 mV·s^-1^ (from inner to outer). The inset is the plot of anodic/cathodic peak currents versus the square root of scan rate**.**

**Figure [S3]** EIS plots of 10 mmoL·L^-1^ [Fe(CN)_6_]3-/^4-^ in 0.1 M KCl that were recorded at cubic SiC NWs electrode and B-doped cubic SiC NWs electrode.

**Figure [S4]** (a) and (b) LSV curves at cubic SiC NWs electrode and B-doped cubic SiC NWs electrode in 0.1 M PBS with 1 mM nitrite at the different scan rates. (c) and (d) Plots of peak current (*Ip*) vs. the square root of scan rate. (e) and (f) Plots of peak potential (*Ep*) vs. the logarithm of scan rate.

**Figure [S5]** The current response to low concentrations of nitrite on B-doped cubic SiC NWs electrode.

**Table S1**. Determination of nitrite at various concentrations in tap water

| **Electrode** | **Sample** | Added (μmoL˙L^-1^) | Found (μmoL˙L^-1^) | Recovery (%) |
| --- | --- | --- | --- | --- |
| **Cubic SiC NWs electrode** | **1** | 10 | 9.863 | 98.63 |
|  | **2** | 5000 | 4935.37 | 98.71 |
|  | **3** | 12000 | 12325.10 | 102.71 |
| **b-doped cubic SiC NWs electrode** | **1** | 1 | 0.9913 | 99.13 |
|  | **2** | 500 | 495.27 | 99.05 |
|  | **3** | 5000 | 4939.15 | 98.78 |
